# Alleviating Promotion of Inflammation and Cancer Induced by Nonsteroidal Anti-Inflammatory Drugs

**DOI:** 10.1155/2017/9632018

**Published:** 2017-05-10

**Authors:** Anthony M. Kyriakopoulos, Markus Nagl, Stella Baliou, Vasilleios Zoumpourlis

**Affiliations:** ^1^Nasco AD Biotechnology Laboratory, 11 Sachtouri Str, 18536 Pireus, Greece; ^2^Division of Hygiene and Medical Microbiology, Medical University of Innsbruck, Schöpfstr. 41, 6020 Innsbruck, Austria; ^3^National Hellenic Research Foundation, 48 Vas. Konstantinou Av., 11635 Athens, Greece

## Abstract

**Clinical Relevance:**

Nonsteroidal Anti-Inflammatory Drugs (NSAIDs) including aspirin are of intensive use nowadays. These drugs exert their activity via the metabolism of arachidonic acid (AA) by cyclooxygenase inhibition. Though beneficial for health in some instances, both unspecific and specific cyclooxygenase inhibitor activity interfere with AA metabolism producing also proinflammatory lipids that may promote cancer.

**Materials and Methods:**

This review is based on available literature on clinical uses, biochemical investigations, molecular medicine, pharmacology, toxicity, and epidemiology-clinical studies on NSAIDs and other drugs that may be used accordingly, which was collected from electronic (SciFinder, Medline, Science Direct, and ACS among others) and library searches of books and journals.

**Results:**

Relevant literature supports the notion that NDSAID use may also promote proinflammatory biochemical events that are also related to precancerous predisposition. Several agents are proposed that may be employed in immediate future to supplement and optimize treatment with NSAIDs. In this way serious side effects arising from promotion of inflammation and cancer, especially in chronic NSAID users and high risk groups of patients, could be avoided.

## 1. Introduction

### 1.1. Inflammation Route via Arachidonic Acid Metabolism

Inflammation is driven by complex metabolic pathways, with arachidonic acid (AA) as one important molecule of origin. AA metabolism is fundamental for both promotion and inhibition of inflammatory processes. Several enzymes are involved in this regulation of inflammation, cyclooxygenases 1 and 2 [1], lipoxygenases [1], cytochrome P 450 (CYP) epoxygenases and *ω*-hydroxylases [2], and also the nonenzymatic processes of AA metabolism like the free radical-catalyzed peroxidation [3]. Nonsteroidal Anti-Inflammatory Drugs (NSAIDs) have been designed to decrease above all the classical symptoms of pain and tumefaction, but in the meantime it is known that they cause proinflammatory effects, too. Aspirin targets the COX-1 pathway, whereas the classical NSAIDs target mainly the COX-2 pathway by inhibiting prostaglandin E_2_ (PGE_2_) formation [4]. The anti-inflammatory effect is due to the inhibition of vasodilatation and to the shortening of mast cell and other immune cells recruitment. Aspirin however acetylates also the COX-2 isoenzyme but due to slight sequence variations this evidently consumes less binding energy for arachidonic acid to become bound and be further metabolized [5].

### 1.2. Cyclooxygenase (COX) Activity

Early findings on aspirin inhibitory mode of action on prostaglandin (PG) synthesis led to the initial discovery of cyclooxygenase (COX) [6, 7]. This enzyme, now called COX-1, is central to AA catabolism to end up producing PGI_2_, also known as prostacyclin, with clear antithrombogenic [8, 9] and cytoprotective to gastric mucosa [10, 11] physiological functions. In 1991, a 64% sequence homologue to COX-1 enzyme was discovered [12, 13] that was inducible in a number of cells to certain proinflammatory stimuli [11] and inhibited in its expression by corticosteroids [7]. This is the enzyme now termed as COX-2. In this area, the variation of severity of side effects caused by different anti-inflammatory drugs including aspirin was puzzling. Particularly, stomach side effects by aspirin led to the development of safer drugs like meloxicam, nimesulide, and etodolac, which are now well accepted as selective COX-2 inhibitors [7]. Although both COX isoenzymes catalyze the oxygenation of arachidonate, COX-2 shows a more diverse substrate selectivity compared to COX-1. For example, COX-2 apart from arachidonic acid oxygenates in the same efficiency 2-arachidonylglycerol (endocannabinoid) [14]. The most evident difference between COX isoenzymes is in their expression in tissue distribution. Unlike COX-1, which is ubiquitous and constitutively expressed throughout the gastrointestinal system, the kidneys, the vascular smooth muscle, and the platelets, COX-2 is constitutively expressed in endothelial cells, brain, and kidneys and is variably induced in its expression by distinct inflammatory stimuli and neoplastic conditions [15, 16]. Unexplained antipyretic and analgesic effects of acetaminophen, phenacetin, and dipyrone, without evident COX-1 or COX-2 inhibition, were made clear by the discovery of yet another COX isoenzyme termed COX-3 that when expressed showed selective inhibition to these agents [17].

### 1.3. How NSAIDs May Cause Side Effects

**Figure 1:**
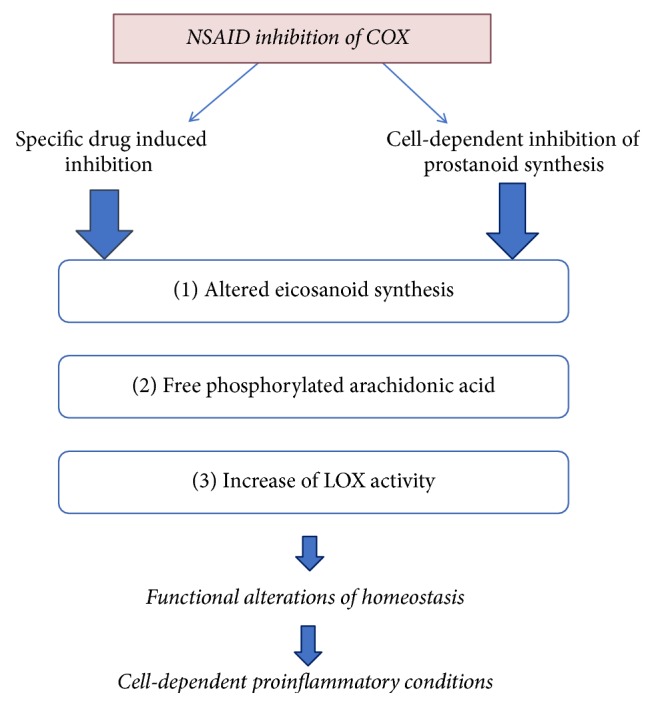
Levels of possible side effects of NSAIDs [18]. Drug- and cell-specific inhibition of COX isoenzymes [15] and respective prostanoids results in alteration of homeostasis [7] and in promotion of proinflammatory conditions [18–22].

NSAIDs, by inhibiting cyclooxygenase enzyme activity, even by different means, may all share to a greater or lesser extent a similar kind of side effects [7]. However, these side effects may be both (a) specific to the NSAID type and (b) cell type specific. Side effects depend on the specific inhibition of prostanoid synthesis due to the agent inducing the COX inhibition and the type of targeted tissue [15]. Prostanoid synthesis alteration contributes to disturbance of homeostasis [7] that may be cell specific, giving an end organ specific toxicity [18–22] (Figure 1).

#### 1.3.1. The Mechanisms by Which Aspirin Induces Proinflammatory Effects

**Figure 2:**
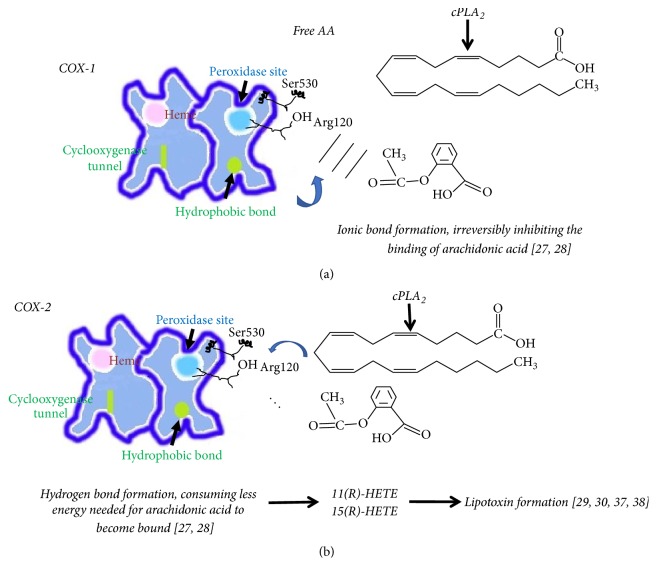
(a) Aspirin acetylation of COX-1 irreversibly inhibits arachidonic acid to become bound, whereas (b) acetylation of COX-2 leads to the formation of lipoxins [27–30, 37].

Acetylation of serine (Ser-530) of COX-1 even by low aspirin concentrations and in a few minutes results in the inhibition of prostaglandin E_2_ (PGE_2_) formation and the inhibition of platelet function (anticoagulant activity) [23]. This acetylating reaction irreversibly inactivates COX-1 activity [24]. As a consequence, related tissue and blood pressure homeostasis depending on PGE_2_ formation may be affected [7, 25]. An example is the kidney normal function that depends on PGE_2_ synthesis. Renin is secreted by PGE_2_ formation and angiotensin II stimulation is mainly mediated by PGE_2_ production by COX-1, but also by COX-2 [25]. Acetylation by aspirin is also occurring on the COX-2 isoform in almost the same manner due to the structural homology between COX-1 and COX-2 isoenzymes. Tyrosine residues (tyr-385) to a greater extent and (tyr-348) to a lesser extent are critical for this acetylation event of COX-2 by aspirin [26]. These tyrosine residues constitute a hydrogen binding network that is critical for the precise positioning and the relative reactivity rendering the closeness of Ser-530 with the acetyl group of aspirin feasible. Arginine 120 (Arg-120) just below Ser-530 in the active sites of COX, however, makes the difference of arachidonic acid binding ability between COX-1 and COX-2 isoenzymes (Figure 2). In the COX-1 case of binding of arachidonic acid, an ionic bond is formed between Arg-120 and the carboxylate of arachidonate. In COX-2 case however, instead of an ionic bond, a hydrogen bond is formed with Arg-120 and arachidonate thus conferring less to the binding energy needed for the molecule to become bound [27, 28]. During the acetylation event in COX-1, the arachidonic acid is irreversibly inhibited from binding when Arg-120 makes the Ser-530 acetylation efficient by forming a weak ionic bond with the carboxylate of aspirin. Conversely, the acetylation event in COX-2 does not irreversibly inhibit its activity but just lowers the arachidonic acid binding ability to the enzyme's active site (Figure 2).

The acetylation event by aspirin, shown to produce also the aspirin-triggered lipotoxins by transcellular (cell-to-cell) interactions [29, 30], is also supported by clinical evidence. Direct clinical evidence shows that among the derived eicosanoids produced by aspirin acetylation are the lipoxin A_4_ (LXA_4_), which is vasodilatory, and the leukotrienes C_4_ and D_4_ (LTC_4_, LTD_4_), which are potent vasoconstrictors. These eicosanoids have been shown to be generated under aspirin treatment in the atherosclerotic lumen of blood vessels [31]. Also, in aspirin intolerance, excessive amounts of LTC_4_ have been isolated from nasal secretions and bronchial biopsies [32]. These leukotrienes produced are implicated to severe gastrointestinal [33] and severe cardiovascular side effects [34] as they constitute important mediators of inflammation, ischemia [35], and bronchoconstriction [36].

#### 1.3.2. The Transcellular Biosynthesis of Eicosanoid Derivatives: Crossover Pathways

**Figure 3:**
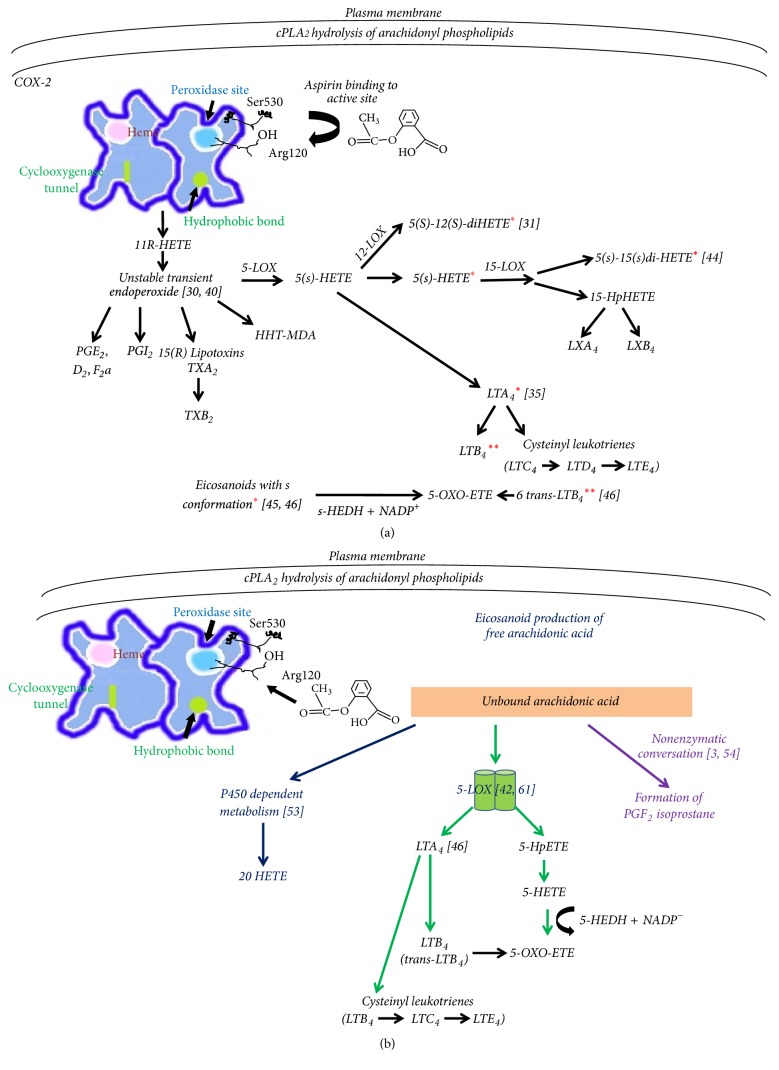
Metabolic events that follow acetylation of COX-2 and further transcellular activities: (a) eicosanoid production by crossover pathways of acetylated COX-2 and LOX isoenzymes [31, 35, 37–44]. (b) Eicosanoid production by free arachidonic acid [3, 44–46, 53, 54, 61].

The transcellular biosynthesis of lipoxins requires interactions between LOX isoenzymes (LOX-LOX interactions) and can promote generation of leukotrienes (LTs) by endothelial cells [37]. When COX-2 is acetylated by aspirin, interaction with 5-lipoxygenase (5-LOX) occurs that triggers the transcellular biosynthesis of 15-R epimers of lipoxins [29, 38]. The derived eicosanoids by the acetylation of COX-2 in close association with 5-LOX are of the type of “S” conformation [39, 40]. Hereby, it has to be emphasized that in all cases arachidonic acid is first transformed to unstable precursor intermediate molecules ending up to many “S” conformations after aspirin treated COX-2 [30, 41]. Therefore, even 15-S-HETE formation cannot be excluded even by aspirin-induced specific 15-R-HETE formation as it may occur also in a nonenzyme dependent fashion [41]. During aspirin-triggered lipoxin synthesis, the precursor LTA_4_ (that is also of “S” conformation) may be formed by 5-LOX and serve as substrate for leukotriene synthesis of LTB_4_ that is formed prior to LTC_4_ and LTD_4_ [35] (Figure 3(a)). Apart from the absolute belief of 15-R-HETE being a sole product, as derived by the acetylation of COX-2, the presence of the double dioxygenated product, 5S-12S-DiHETE isolated in vivo, suggests further transcellular metabolic events that show further eicosanoid synthesis by 5- and 12-LOX interactions [31]. Research during that time may have identified generation of byproducts via enzymatic conversion of LTA_4_ [30, 42, 43]. Similar latter results indicate the possibility of 5S-15S-DiHETE to be formed in vivo by the acetylated COX-2 activity and 5-LOX-15-LOX interactions having 5S-hydroxy-6E,8Z,11Z,14Z-eicosatetraenoic acid (5-S-HETE) as a substrate [44]. As LOX activity is not inhibited by aspirin, through the unstable intermediate formed by the acetylated COX-2, LOX isoenzymes continue the eicosanoid synthesis to produce active compounds [30, 40]. Further, all S-conformations produced during COX-2 acetylation and interaction with lipoxygenases can be relatively good substrates for 5-hydroxyeicosanoid dehydrogenase (5-HEDH) to produce 5-OXO-ETE [45]. Thus, another important proinflammatory mediator may be also formed (Figure 3(a)). Under oxidative conditions 5-HEDH transforms (5-S-HETE) to 5-OXO-ETE [46] as under normal conditions 5-HEDM is inactive because it requires NADP^+^ as a cofactor. Under stress conditions, however, NADPH oxidase activity has been described for its serious role in mediating inflammation [47].

5-S-HETE is on its own a potent proinflammatory mediator [48] to induce stress conditions together with potent proinflammatory leukotrienes LTB_4_ and the cysteinyl leukotrienes LTC_4_ and LTD_4_. The metabolic tendency of 5-LOX to produce the 5-OXO-ETE derivative coupled with LTB_4_ is important for inflammation and cancer [48, 49], which has not been linked adequately as a serious proinflammatory condition due to NSAID use. The 5-S-HETE metabolite, when accumulating, is a potent neutrophil activator [50]. The 5-OXO-ETE derivative, however, has been shown to be a 100 times more potent neutrophil activator than its precursor [51]. As synthetic 5-OXO-HETE derivatives prove to be even more potent than 5-OXO-ETE, the native derivatives also traced in vivo may be further implicated in the promotion of chronic inflammation and cancer [49, 52]. 5-OXO-HETE acts proinflammatorily via the OXO-ETE receptor that is known to promote eosinophil and other inflammatory cells migration [48]. 5-OXO-ETE is formed in neutrophil microsomes under the presence of NADP^+^- and Ca^2+^- dependent translocation of 5-LOX to the nuclear membrane to act on arachidonic acid bound by the nuclear membrane accessory protein 5-LOX activating protein (FLAP). The neutrophil microsomes under reduced conditions prefer, as well as the 5-S-HETE, to produce the 5-OXO-ETE derivative lipid using the 6-transanalogue of LTB_4_ as a substrate [46].

Free activated arachidonic acid not bound to COX-2 may be also used by 5-LOX to produce leukotrienes during inflammation in vivo [42]. Furthermore, it may be utilized by the P450 metabolism to produce 20-HETE [53] and by nonenzymatic conversion to form PGF_2_ isoprostanes [3, 54] (Figure 3(b)).

## 2. Materials and Methods

The literature study was conducted from scientific journals and books and electronic sources such as SciFinder, Science Direct, Medline, and Google Scholar, covering the period from January 1945 to the end of December 2016.

## 3. Results

### 3.1. Nonaspirin NSAIDs

#### 3.1.1. Traditional NSAID Clinical Side Effects

Traditional NSAIDs during clinical practice vary on the degree of causing vascular side effects. Increased risk is noticed by high doses of diclofenac and ibuprofen due to the increased myocardial infarction events recorded, whereas increased doses of naproxen have substantially smaller risk [55], suggesting differential inhibition of activity of COX-2. Acute myocardial infarction risk is potentiated in patients with coronary artery disease by high and low doses of diclofenac and rofecoxib and other NSAIDs, but not with naproxen even when administered in high doses [34]. The risk for renal disease development is tightly correlated with acetaminophen overuse [56]. Acetaminophen provides a unique example of cell-specific COX inhibition that may negatively affect the prostanoid synthesis in tumor cells by altering the levels of PGE_2_ [57, 58]. Upon NSAID inhibition of COX activity by traditional COX-2 inhibitors like diclofenac, an alternate housekeeping COX-1-like activity, of a third COX isoenzyme may be also inhibited by concurrent use of acetaminophen [28]. Also, this drug's specific COX-2-like inhibition may affect homeostatic mechanisms of the central nervous system, the gastrointestinal system, and the renal system [15].

#### 3.1.2. Proinflammatory Mechanisms Caused by Traditional NSAIDs

In general, for traditional nonselective COX inhibitors, the mechanism of drug generated myocardial pathology [34] may be due to prostacyclin and other prostanoid inhibition that depends on the degree of COX-2 inhibition [7]. The constitutive COX-2 isoenzyme that is inhibited plays an important role in the regulation of salt, volume, and blood pressure maintenance [59] by providing the appropriate prostaglandins to regulate the renin-angiotensin system [60]. Apart from prostanoid synthesis diminishment, free hydrolyzed arachidonic acid from c Phospolipase A_2_ (cPLA_2_) may be utilized by LOX isoenzymes to produce increased amounts of proinflammatory leukotrienes [61] and toxic metabolites like 5-S-HETE and 5-OXO-ETE as seen with the aspirin acetylation of COX-2 [45, 46] (Figure 3(b)).

### 3.2. “More Selective” COX-2 Inhibitors

#### 3.2.1. Proinflammatory Mechanisms by “More Selective” COX-2 Inhibitors

The clear distinction between COX-1 and COX-2 inhibitors cannot be defined fully [62]. The term selective COX-2 inhibitor requires further examination as it is oversimplified and therefore the term “more selective” is used in this article. To identify proinflammatory effects caused by COX-2 more selective inhibitors, the interrelationships between COX-1 and COX-2 catalytic functions have to be taken into account. COX-1 and COX-2 have similar binding sites for NSAIDs other than aspirin that block arachidonic acid metabolism. Naproxen, for example, due to its smaller molecular size occupies easily the hydrophobic COX-1 binding site of arachidonic acid where an isoleucine is at position 523. Celecoxib is a larger molecule that naproxen cannot occupy the COX-1 binding site for arachidonic acid. Instead it occupies in an easier manner the COX-2 binding site where a smaller valine instead of isoleucine is at position 523 [63]. Thus due to small structural differences between the two COX isoenzyme active sites NSAIDs show greater or lower selectivity for COX-1 and COX-2 resulting in greater or lower relative inhibition of arachidonic acid metabolism. Relatively increased inhibition of COX-2 activity results in relative diminishment of prostacyclin inhibition which is a known cardioprotective prostanoid [64]. Recent epidemiologic studies come to directly associate the use of a large number of individual NSAIDs with hospital administration for heart failure [65]. Looking at a different metabolic pathway, more selective COX inhibitory NSAIDs may block the metabolism of 20-HETE to PGF_2*α*_ and other mediators during P450 metabolism of arachidonic acid thereby resulting in increased accumulation of 20-HETE [53] (Figure 3(b)). 20-HETE has also been shown to be a serious promoter of renal hypertension and to be implicated in an increased risk for renal [33] and cardiovascular diseases [34] such as myocardial infarction, hypertension, and heart failure that have also been observed but in a smaller scale with the administration of nonaspirin traditional NSAIDs [6, 7, 34] (Figure 3(b)). Arachidonic acid that remains not bound and oxygenized by COX isoenzymes may be used by p450 and 5-LOX dependent, as well as enzyme independent, metabolic pathways to produce proinflammatory metabolites [45, 46, 61] like LTB_4_, LTC_4_, and 5-OXO-ETE as in aspirin acetylation's case (Figure 3(b)).

#### 3.2.2. “More Selective” COX-2 Inhibitor Clinical Side Effects

Withdrawal of rofecoxib (a similar agent to celecoxib with increased vascular side effects) from clinical use is perhaps the best example to account for side effects by a more selective COX-2 inhibitor [53, 66]. Clearly, increased myocardial infarction events are associated with more selective COX-2 inhibitor use although nonselective inhibitors of cyclooxygenase are not excluded from being potent risk factors for the development of cardiovascular episodes [34, 62, 67].

Coadministration of aspirin in clinical practice is recommended for certain groups of patients taking vast amounts of NSAIDs as a thrombolytic agent for cardioprotection [63]. These patients are at increased risk from thrombotic events by taking selective COX-2 inhibitor NSAIDs to treat inflammatory disorders [68]. Guidelines however state that aspirin use may not always be an efficient protection [62]. As for traditional NSAIDs, the more selective COX-2 inhibition may also contribute to a subsequent surplus of arachidonic acid that can be used by lipoxygenases (Figure 3(b)). As already described 5-LOX may be an important mediator enzyme for inflammation and cancer producing more proinflammatory leukotrienes LTC_4_ [69], LTB_4_ [46, 61, 70], and 5-OXO-ETE [45, 49].

### 3.3. Hypersensitivity Response

Eosinophils among other stimuli are also driven by LTC_4_, which is induced by NSAID use (Figures 3(a) and 3(b)), and are essential mediator cells in the production of allergic inflammation [71]. Various types of NSAIDs are warranted for causing respiratory intolerance [72]. By proinflammatory mediator generation they have been implicated to produce allergic and inflammatory reactions as well as ischemia at the level of lung mucosa leading to asthma [73, 74]. NSAID-induced gastrointestinal injury is mediated by increased LTB_4_ synthesis, too. LTB_4_ level is elevated in arthritis treated with NSAIDs [75] (Figures 3(a) and 3(b)). Indomethacin may cause acute gastropathy, and the induced overproduction of tumor necrosis factor *α* (TNF*α*) has also been implicated in the pathogenesis of disease state [76]. Complementarily, indomethacin to a greater extent than ibuprofen causes renal dysfunctional abnormalities in preterm neonates, and unfortunately both are the drugs of choice for patent ductus arteriosus failure [77]. The immune response in urticaria provides another good example for NSAID driven side effect [78]. Urticaria is the clinical term of a heterogenous group of diseases characterized by wheels and flares of skin's vascular inflammation. Aspirin and other more selective to COX-2 NSAIDs (rofecoxib) and traditional NSAIDs (nimesulide, acetaminophen) cause the aspirin acute intolerant urticaria that in some cases may lead to the aspirin chronic urticaria [79] that has a sound basis of autoimmunity [78]. As it has been shown that in chronic cases of urticaria a specific set of IgE autoantibodies directed against thyroid peroxidase may constitute a novel pathogenetic mechanism, this may be serious for chronic NSAID users [80]. Selective to COX-2 NSAIDs and aspirin have been reported to be implicated in hypersensitivity responses and excess of histamine release, and this may be extended to various hypersensitivity immune disorders [81–83]. However, further studies are needed to investigate the possible IgE elevation in urticaria events that is caused by “more selective” NSAIDs.

## 4. Discussion

### 4.1. Alleviating NSAID Associated Proinflammatory Activity

As already described, NSAID side effects occur primarily due to the inhibition of cyclooxygenases that metabolize arachidonic acid and synthesize prostaglandins with beneficial activities under normal conditions [7, 62]. An example of a current therapeutic way to overcome NSAID side effects is the combination of aspirin coadministration in patients receiving “more selective” COX-2 inhibitors in order to avoid thrombotic vascular events, although this may not always be sufficiently protective [62]. Scientific evidence remains to be clarified by large epidemiological and meta-analysis studies to establish safety standards for patients in high risk of developing serious cardiovascular side effects [84]. This process is both time and cost demanding. For example, results from large and recent epidemiologic studies in Europe clarify that heart failure is associated with increased NSAID usage. This increased risk of heart failure is dose dependent and associated with traditional and more selective to COX-2 inhibitors [65]. In all respects, the optimum selection of NSAID coadministration requires deep scientific knowledge to identify the bottom end of prostaglandin synthesis and inhibition with subsequent imbalance of homeostasis (Figure 1).

Better chances to optimize treatment of NSAIDs with relatively high and low COX-2 inhibitory activities can perhaps be conferred by supplementary agents that may interfere with COX in a different manner.

Arachidonic acid hydrolyzed by cPLA_2_ (phospholipase A_2_), if not metabolized by cyclooxygenases, remains an available substrate to be used in other catabolic pathways: (1) the lipoxygenase, (2) the P450 epoxygenase, and (3) the nonenzymatic synthesis leading to isoprostane (Figure 3(b)).

It has been described that by inhibition of COX activity the increase of cysteinyl leukotriene family (CysLT) potent proinflammatory lipid mediators is feasible [63]. Human studies on aspirin intolerance support this hypothesis. When PGE_2_ levels are decreased by inhibition of COX-1, altered prostanoid production, combined with increased enzymatic expression like the LTC_4_ synthase expression, leads to increased leukotriene synthesis producing the disease state [32, 85, 86].

A way to circumvent proinflammatory leukotrienes (LTB_4_ and LTC_4_) production by an overwhelming 5-LOX activity may be the already developed specific LOX inhibitors [87, 88]. These may block the undesirable side effects of both LTB_4_ and LTC_4_ (a cysteinyl derived leukotriene) [89]. Furthermore, the use of selective agonists of cysteinyl leukotriene receptor 1 (CysLTR_1_) is referred to circumvent leukotriene-associated pathologies probably by inhibition of cytosolic Ca^2+^ [90]. Also the inhibition of CysLTR by other agents may provide suitable pharmacologic activity. The use of either leukotriene biosynthesis inhibitors or leukotriene receptor antagonists [91] may also help to minimize NSAID side effects. Since LTC_4_ production is blocked by inactivation of CysLT_1_ receptor, selective CysLT_1_ antagonists may be applied [92]. Also, CysLT_1_ receptor antagonists to reduce eosinophilia may be of therapeutic value [91].

NSAIDs activity as already discussed (Figures 3(a) and 3(b)) may favor 5-LOX to catalyze the formation of LTA_4_ and 5-S-hydroperoxy-6,8,11,14-eicosatetraenoic acid (5-HpETE), which is rapidly reduced to 5S-hydroxy-6E,8Z,11Z,14Z eicosatetraenoic acid (5-S-HETE). This metabolic intermediate is oxidized by 5-hydroxyeicosanoid dehydrogenase (5-HEDH) to produce 5-OXO-ETE [46]. 5-HEDH activity is inhibited by 5-hydroxy-fatty acids [45]. Recent advances in the formation of 5-OXO-ETE receptor antagonists [93] may also help to prevent hypersensitivity reactions by COX inhibitors. Conversion of 5-S-HETE into 5-OXO-ETE is highly reversible in the presence of NADPH and alleviation of oxidant conditions. The use of antioxidants may favor restoration of 5-OXO-ETE side effects via the 5-OXO-ETE receptor in asthma, cancer, and cardiovascular conditions [48, 94].

Apart from LOX favored metabolism during COX inhibition, oxidized arachidonic acid may sustain nonenzymatic conversion to form prostaglandin F_2_ compounds (PGF_2_-isoprostanes). Isoprostanes are very readily formed in biological fluids [3] from oxidized arachidonic acid and through endoperoxide intermediates [54]. Modulation of inflammation by NSAIDs as a natural way of treatment is a very natural way of treatment is by the concurrent prolonged use of naturally derived d-*α* tocopheryl acetate [95, 96]. Asthmatic and atherosclerotic patients seem to benefit by natural-source d-*α*-tocopheryl acetate as this is shown to reduce allergen-induced F_2_-isoprostane formation [95, 97].

Arachidonic acid, once liberated from membrane phospholipases and not being metabolized further by cyclooxygenases due to NSAID inhibition, may be efficiently metabolized by isoforms of the cytochrome P450 (CYP) family to form 20-hydroxyeicosatetraenoic acid (20-HETE) [98, 99]. 20-HETE promotes coagulation of platelets, thus shortening the time of bleeding, and its synthesis is being increased by rofecoxib, suggesting serious cardiovascular side effects for this drug [53].

In order to identify agents that may inhibit undesired 20-HETE synthesis by NSAID-COX inhibition, the experimental model of spontaneous hypertensive rats provides significant clinical information. Agents that induce heme oxygenase reduce the renal formation of 20-HETE and also decrease hypertension [100, 101]. In clinical research, heme oxygenase inducers are of increasing interest to overcome spontaneous reactions that lead to kidney failure [102].

Heme oxygenase-1 (HO-1), which is expressed in all tissues, receives electrons from NADPH by P450 enzyme fractions due to CRP microsomal protein mediator and P450 protein-to-protein interactions [103]. This may prove to be important clinically, since under severe hypoxia there may be a way to circumvent 5-OXO-ETE accumulation by expenditure of NADPH to NADP^+^ to reform 5-HETE [104] and to deprive arachidonic acid reserves to form 20-HETE by P450 enzymes at the same time [105] (Figure 6). In this respect, attention is drawn on the induction of HO-1 by naturally derived agents like the endogenous haloamines of taurine, that is, N-chlorotaurine (NCT) and N-bromotaurine (NBrT), also termed as small molecule NSAIDS [106, 107]. These haloamines have been shown to downregulate the production of Cox-2 derived PGE_2_ [108] in a way independent of COX-2. NCT exerts its anti-inflammatory activity in rheumatoid arthritis synoviocytes by inhibiting IL-*β* induced production of PGE_2_ by decreasing COX-2 isoenzyme expression leaving COX-1 expression unaltered [109]. However, at lower cytotoxic concentrations both NCT and NBrT decrease PGE_2_ synthesis without affecting COX-2 expression [108]. Haloamines of taurine (NBrT and NCT) at present state can be administered locally in cases of cutaneous body cavities and organ infection and inflammation to inactivate microbes, minimize inflammation, and reduce pain and other symptoms [110–113].

**Figure 4:**
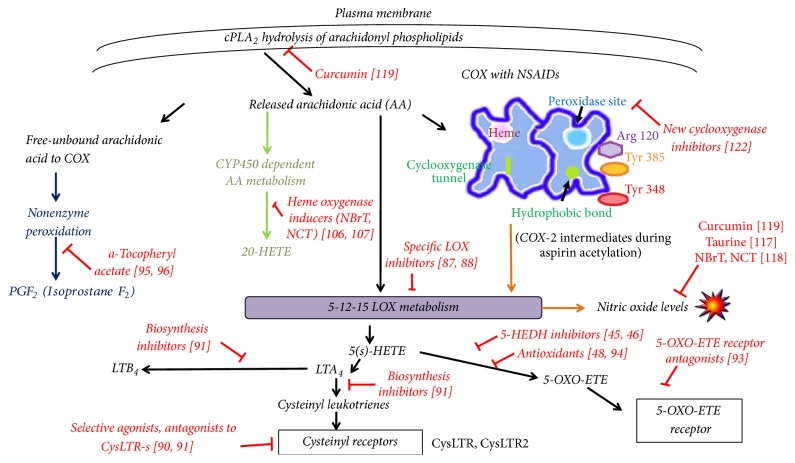
Possible target points of supplementary agent's use to alleviate NSAID promotion of proinflammatory and cancerous conditions.

Another target for NSAID minimization of side effects on the cardiovascular system may be the maintenance of low levels of nitric oxide (NO) that are essential for cardioprotection [114] (Figure 4). NO at normal levels inhibits thromboxane synthase and activates prostacyclin synthase [115]. LOX activity leading to increased LTB_4_ and LTC_4_ formation may create a surplus of reactive oxygen species (ROS) and especially superoxide [63]. In such a case, cardioprotective levels of nitric oxide may be consumed by ROS to form peroxynitrite, a prostacyclin synthase inhibitor and thromboxane receptor stimulator [116]. The overall effect caused by more selective COX-2 inhibitors is a low level of prostacyclin and high levels TXA_2_ [63], promoting a platelet activating thrombosis event. Restoration of nitric oxide levels is said to be achieved by consumption of certain doses of taurine (2-aminoethanesulfonic acid), which may act as an antioxidant on a diseased vascular state and as a prooxidant in an otherwise normal vasculature being at risk from NSAID use [117]. Also, taurine derivatives NCT and NBrT are known to reduce excessive nitric oxide formation [118]. Curcumin, a natural antioxidant consumption, may also be of help for NSAID users, especially for those being at risk. Curcumin modulates arachidonic acid release from cellular membranes by blocking the hydrolysis event by cytosolic phospholipase A_2_, and it inhibits the 5-LO catalytic functions and nitric oxide synthase activity [119]. During COX-1 and COX-2 inhibition by NSAIDs, generation of NO and ROS is not suppressed under inflammatory stimulation, whereas cPLA_2_ activity is increased under inflammatory conditions. A probable direct synergy with its function with 12- and 15-LOX isoenzymes to produce NO and ROS, via a cPLA_2_. 12- and 15-LOX pathway are suggested [120]. The use of 12-/15-LOX inhibitors may be beneficial, especially in neurodegenerative diseases where NO activity is a major proinflammatory mediator [120, 121].

Finally, recent scientific effort is focusing on the trials of new cyclooxygenase inhibitors [122] in order to overcome undesirable cyclooxygenase metabolism of arachidonic acid in inflammation and cancer. These compounds have lower isoform selectivity to COX than the NSAID “more specific” cyclooxygenase inhibitors (coxibs), which may result in reduced side effects.

### 4.2. NSAIDs and Risk for Cancer

#### 4.2.1. Studies with Clinical Evidence for Cancer Development

Epidemiologic studies provide contradictory results on cancer risk development by NSAIDS that may be due to the cell-specific activity of producing prostanoids and the specificity on COX inhibition by a particular NSAID (Figures 1 and 2 and Table 1).

Subjects using certain types of NSAIDs are being protected from colorectal cancer [123]. Specifically, aspirin users have better protection from the close to rectum and distal to colon cancer development, whereas the nonaspirin NSAID users are being more protected from the proximal to colon cancer development [123–125]. It should be mentioned, however, that anatomic locations in this type of cancer development are also tightly associated with the age, gender, and the race of patients [126]. Some studies indicate that breast cancer development risk is also lowered by NSAID use [127, 128], although this may be a small decrease of relative risk [129]. Moreover, type of NSAID, specific dose, and duration of treatment have not been yet identified [130]. Other studies, however, indicate increased risk for developing breast cancer irrespective of hormonal status (estrogen/progesterone (ER/PR) receptor positive/negative) and that the risk for developing ER/PR (−) breast cancer is raised in long term daily use aspirin users [131, 132]. Traditional NSAID use (diclofenac, etodolac, and meloxicam) is also associated with decreased risk in developing aggressive skin cancers, whereas “more selective” to COX-2 inhibitors were not found to be protective [133, 134]. Also, whilst aspirin and other NSAIDS may protect from developing esophageal and noncardia gastric carcinomas, these are nonprotective for the development of cardia gastric carcinoma [135, 136].

Epidemiological studies are controversial regarding a protective [137] or promoting [138] effect of NSAIDs for prostate cancer. In prostate cancer, genetic variation in the COX-2 gene is associated with increased risk [139]. Epidemiologic studies clearly indicate that acetaminophen and nonaspirin NSAIDs are associated with a significant risk of developing kidney cancer [140, 141]. Complementarily, whilst aspirin and other NSAID users have lower risk for developing hepatocellular carcinoma (HCC) [142, 143], men only gain protection from intrahepatic carcinoma (IHC) by taking aspirin and this did not account for any other NSAID use [142]. Aspirin and other NSAID users are not protected from developing brain tumors and the use of traditional and “more selective” COX inhibitors seems to increase the risk for developing meningiomas [144].

Although epidemiology data on cancer risk by NSAIDs are controversial, by comparison of results important indications may be drawn (Table 1). Inhibition of prostanoid synthesis due to aspirin acetylation on COX isoenzymes, although in a different manner [7, 26], may be protective of a variety of types of cancers. Acetylating of COX by aspirin was found to be protective of prostate cancer development in a subgroup of subjects having specific sequence variations within the COX-2 genome [139]. Also, risk reduction for colorectal cancer development with aspirin is related only to specific genotypes near the IL-6 genome [145]. Thereby genetic variation seems be an important parameter for the aspirin effect in cancer development. By contrast, aspirin use increases the risk for breast cancer development irrespective of hormonal influence [131]. Cell-dependent prostanoid formation is also another important serious parameter (Figure 1). Any given prostanoid forming cell selects a particular prostanoid as its major product [15]. Brain and mast cells, for example, preferably produce PGD_2_, and its formation provides the core for vital homeostasis mechanisms [146, 147].

#### 4.2.2. What Are the Effects of NSAIDs on Cancer?

Traditional and “more selective” COX inhibitors preferentially bind on arachidonic acid's active site of respective isoenzymes. The degree to which COX inhibitors cause inhibition of COX-1, COX-2, or COX-3 depends on their selective preference for COX active sites [62, 63]. Under normal conditions, constitutive COX-1 maintains prostacyclin and PGE_2_ levels as well as other prostanoids in all tissues, whereas COX-2, when activated in an inducible way (i.e., during inflammation), produces prostacyclin and other prostanoids in most, but not all, organs [15]. From epidemiologic studies it is evident that some groups of subjects benefit from developing certain types of cancer by nonaspirin NSAIDs, for example, nonmelanoma skin cancers [134], whereas the same use of NSAIDs elevates the risk of developing some other types of cancer, for example, in the kidney [141]. The differential inhibition of COX-1, COX-2, and COX-3 isoenzymes results in differential beneficial activities and side effects [15] (Figure 1). For cancer, the situation is even more complicated as it may also depend on both the impact on COX and the subsequent synthesis inhibition of protective prostaglandins.

### 4.3. Mechanisms of Promotion of Cancer by the AA System and the NSAIDs

PGE_2_ association with tumorigenesis has been thoroughly investigated. Measurement of increased amounts of PGE_2_ in colorectal cancer has long been implicated to contribute to tumorigenesis [148], where apparently COX-2 and not COX-1 is overexpressed [149], and COX-2 expression is more essential for tumor development in the distal colon [150]. Nowadays, inducible PGE_2_ synthesis is implicated in many types of cancers [58, 151]. However, it should always be considered that PGE_2_ is a major component of tissue homeostasis under normal conditions [21, 58] and its constitutive synthesis may be impaired by the frequent use of all classes of NSAIDs [7].

**Figure 5:**
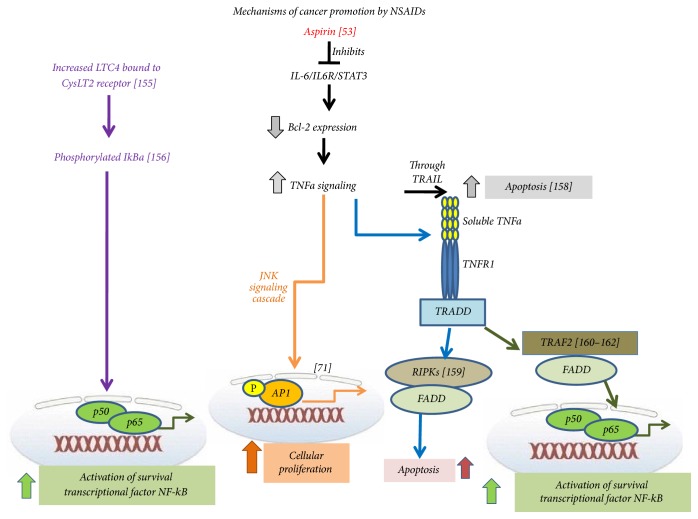
Molecular pathways that may contribute to promoting cancer by NSAID use [155–158, 160–163]. LTC_4_ and TNF_a_ may activate transcription factor NF-kB and increase cellular proliferation by concurrent pathways that otherwise induce apoptosis.

Frequent use of NSAIDS leading to depletion of COX activity may favor the metabolism of arachidonic acid by the LOX pathways [15, 63] as discussed. There are several human and animal studies finely reviewed to support this hypothesis [18]. 5- and 12-LOX metabolic pathways are linked to carcinogenesis [152], and altered COX and LOX metabolism of arachidonic acid are a common factor in malignancy [35]. One major metabolite of 5-LOX, the LTB_4_, was shown to produce cancer predisposition by activating transcriptional factor NF-kB in hepatoma cells [153]. The cysteinyl leukotrienes (TLC_4_, LTD_4_, and LTE_4_) may also be oversynthesized as increased amounts of LTC_4_ and LTD_4_ and decreased amounts of PGE_2_ are detected in nasal secretions if patients with aspirin intolerance are treated with this medication [154] (Figure 3(a)). LTC_4_ induces the phosphorylation of NF-kB p65, activates the complex NF-kB p50-p66 [155], and via the CysLT2 receptor induces the phosphorylation of IkBa by involving protein kinase family enzymes [156]. Aspirin decreases the expression of Bcl-2 by blocking the IL6-IL6R-STAT_3_ signaling pathway [157]. The decreased expression of Bcl-2 may cause apoptosis by tumor necrosis factor apoptosis-inducing ligand and increased levels of TNF*α* expressed [158]. However, overproduced TNF*α* may not function proapoptotically but contribute to cell survival (Figure 5). TNF*α* bound to the tumor necrosis receptor 1 (TNFR-1), apart from other causalities, recruits TNFR-1-associated death domain protein (TRADD). TRADD in turn has a dual activity. When TRADD recruits the receptor interacting protein kinase- (RIPK-) and Fas-associated death domain protein (FADD), this finally results in apoptosis [159]. However, when TRADD recruits TNF receptor factor 2 (TRAF-2) and FADD, this results in activation of survival transcription factor NF-kB [160–162]. TNF-*α* also results in activation of the transcription factor AP-1 via the JNK signaling cascade, which subsequently increases cellular proliferation [163].

Furthermore, extensive 5-LOX activity from arachidonic acid accumulating from NSAID inhibition of COX may also lead to increased 5-OXO-ETE formation (Figures 3(a) and 3(b)). This may be of raised interest in oncology studies as 5-OXO-ETE lipid molecules seem to be required for cancer cell proliferation [48]. In prostate cancer cells, for example, 5-OXO-ETE and the 12-LOX metabolism are also important for tumor propagation [48, 88]. Marked expression of 5- and 12-LOX is being detected in prostate neoplasia in contrast to normal and benign epithelia [88]. The platelet 12-LOX overexpression in prostate cancer is also said to be a trigger for angiogenesis and tumor growth by enhancing av*β*3 and av*β*5 integrin expression [15]. Complementarily, by data drawn from a Gln261Arg polymorphism of the 12-LOX gene meta-analysis study, clearly enough, this polymorphism was shown to be a significant risk factor for increased susceptibility to at least five types of cancer, including prostate cancer, specifically in the Asian population [164].

### 4.4. Description of New Drugs and Their Possible Use for Alleviation of Cancer Predisposition with NSAIDs

**Figure 6:**
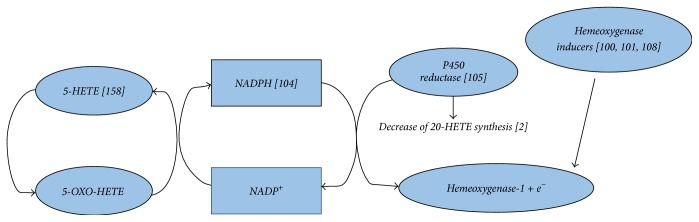
Proposed mechanism of heme oxygenase inducer application to overcome accumulation of toxic metabolites [102, 104, 105] induced by NSAIDs. Induction of heme oxygenase by several agents [100, 101, 108] and receival of electrons via P450 [103, 105] may result in decreased formation of 20-HETE and 5-OXO-ETE toxic metabolite accumulation.

Lipoxygenases are an emerging group of cancer targets as numerous studies indicate that 5-LOX and 15-LOX-1 are associated with the development of cancer via the NF-kB pathway [61]. Treatment with specific LOX inhibitors may be important as therapeutic option in order to overcome LOX overexpression [88]. Looking at the pathway of downstream production of 5-OXO-ETE, as a serious promoter of carcinogenesis, the novel synthesis of 5-OXO-ETE receptor antagonists may be another therapeutic option [93] (Figure 5). Recently we have discovered that the taurine derivative NBrT is a significant proliferative inhibitor leading to cell death among numerous cancer cell lines. Antiproliferative activity is enhanced on PC3 (prostate cancer), A549 (lung cancer), HeLa (cervical cancer), and MDA-MB231 (breast cancer) cell lines, (A. Kyriakopoulos et al. unpublished data). It would be a future interesting model to test the aspirin-induced proliferative ability of these particular cell lines and subsequently the inhibitory effect by the heme oxygenase inducers such as NBrT. The extensive antiproliferative effect on numerous human cancer cell lines by NBrT is in accordance with the recently demonstrated effect of G cycle arrest of glucocorticoid resistant cancer cells and the optimized concurrent anticancer effect of cisplatin with NBrT. NBrT has been considered in studies as a small molecule NSAID as it leads to decreased PGE_2_ levels independently of COX expression [106, 108, 165]. As cyclooxygenase [166] and lipoxygenase [61] metabolism have long ago been associated with tumorigenesis, a possible therapeutic intervention with heme oxygenase inducers (including taurine derivatives) may be of significance and should be tested in animal models. Stress conditions that may lead to renal disease [33, 53, 103] (under hypoxic conditions [60]) and possible cancer predisposition enhancement may be circumvented (Figure 6).

## 5. Conclusion

Only scarce previous studies in the past have been focused on the avoidance of adverse effects of NSAID use [167]. Due to both older and recent research data on proinflammatory effects and cancer development in connection with NSAIDs, selective therapeutic targets and newer agents like the small molecule NSAIDs with an improved benefit-risk ratio become of interest. By all means, a thorough investigation of lipid metabolism under NSAID use is required, and this must be coupled with large scale epidemiological studies to provide valuable clinical information. For example, although accumulated data suggest a protective role of some COX inhibitors in the development of certain types of cancer, the predicted increased risk for other cancers by NSAID use is also equally important. Alterations of AA metabolism by NSAID, prompt further investigating the possible development of inflammation and cancer.

## Figures and Tables

**Table 1:** Cancer risk development from NSAID use as recorded from various epidemiology studies.

Type of cancer	Aspirin	Traditional nonaspirin NSAID	More selective COX-2 inhibitor
Proximal colon^*∗*^	No effect on risk [123] Decreased risk in women [124]	Reduced risk [123]	No data available (NDA)

Distal colon^*∗*^	Decreased risk [123] No effect in women [124]	Reduced risk [123]	NDA

Rectum^*∗*^	Decreased risk [123] No effect in women [124]	No effect on risk [123]	NDA

Nonmelanoma skin cancer (NMSC)	Decreased risk for BCC^a^ SCC^b^ [133] Slightly decreased risk for BCC SCC [134]	Decreased risk for BCC SCC [133] Slightly decreased risk for BCC SCC [134]	Decreased risk for BCC SCC [133] Stronger decreased risk for BCC SCC [134]

Melanoma skin cancer (MSC)	Slightly decreased risk [133]	Slightly decreased risk [133]	No effect on risk [133]

Breast ER/PR (+)	Highly increased risk by aspirin-only NSAID users [131] No effect on risk [127]	No effect on risk by acetaminophen [131]	Increased risk [131] Decreased risk in HER2+ [127] ^*∗∗*^

Breast ER/PR (−)	Highly increased risk by aspirin-only NSAID users [131] Increased risk [132]	No effect on risk by acetaminophen [131]	Increased risk [131] Decreased risk in HER2+ [127] ^*∗∗*^

Brain glioma	No effect on risk [144]	No effect on risk [144]	No effect on risk [144]

Brain meningioma	No effect on risk [144]	Slightly increased risk [144]	Slight increased risk [144]

Hepatocellular Carcinoma (HCC)	Decreases risk [142, 143]	Decreases risk [142]	Decreases risk [142]

Intrahepatic cholangiosarcoma (ICC)	Decreases risk in men [142] No effect on risk in women [142]	No effect on risk by ibuprofen [142]	No effect on risk [142]

Prostate^*∗∗∗*^	No effect on risk [138]	Increased risk with acetaminophen, even stronger for metastatic type [138]	Increased risk, even stronger for metastatic type [138]

Esophageal squamous cell carcinoma (ESCC)	Decreased risk [135, 136]	Slightly decreased risk [135, 136]	Slightly decreased risk [135, 136]

Esophageal adenocarcinoma (EA)	Decreased risk [135, 136]	Slightly decreased risk [135, 136]	Slightly decreased risk [135, 136]

Noncardia gastric carcinoma	Decreased risk [135, 136]	Slightly decreased risk [135, 136]	Slightly decreased risk [135, 136]

Cardia gastric carcinoma	No effect on risk [135, 136]	No effect on risk [135, 136]	No effect on risk [135, 136]

^*∗*^Anatomic locations are associated with gender, age, and race of patients [126]. ^*∗∗*^Study indicates decreased risk in special subgroups of patients. ^*∗∗∗*^Genetic predisposition may increase risk [139]. ^a^Basal cell carcinoma. ^b^Squamous cell carcinoma.
